# Prevalence of malaria and quantification of cytokine levels during infection in East Nile locality, Khartoum State: a cross-sectional study

**DOI:** 10.12688/f1000research.19217.2

**Published:** 2021-08-16

**Authors:** Hwida Barkat, Ahmed Bakheet Abd Alla, Ahmed Galander, Tagwa Salah, Tayseer Elfaki, Ali Nasir

**Affiliations:** 1 Laboratory Administration, Ministry of Health, Sudan, East Nile Locality, Khartoum, Sudan; 2 Parasitology and Medical Entomology, College of Medical Laboratory Science, Sudan University of Science and Technology, Khartoum, Sudan

**Keywords:** cytokines, IL – 10, TNF-α, IFN-γ, recurrent, malaria, Plasmodium

## Abstract

**Background: **The cytokines interferon gamma (IFN-γ), interleukin-10 (IL-10) and tumor necrosis factor-α (TNF- α) play an important role in malaria infection. The aim of this study was to determine the prevalence of malaria and to evaluate cytokine responses to malaria infection in patients from the East Nile locality of Khartoum State.

**Methods:** This study was carried out from May to July 2018 in the East Nile Locality, Khartoum State. Blood samples were collected from 384 randomly selected patients for blood film analysis. Of these, 39 were selected for cytokine level analysis (10 control and 29 patient samples), determined using enzyme-linked immunosorbent assays.

**Results: **The malaria prevalence rate among 384 patients was 18.5%. *Plasmodium falciparum *was the most prevalent (13%), while the prevalence of *Plasmodiumvivax *was 4.6%. The rate of mixed infection was 0.8%. There was a higher prevalence rate (22.7%) in males than females (15.6%). However, we found no significant correlation between cytokine levels and parasitemia in the study group. Nevertheless, our study demonstrated a significant correlation between cytokine levels and recurrent infections.

**Conclusions: **Together, our data show that malaria remains a public health problem in East Nile locality with a high prevalence. Additionally, serum levels of IFN- γ and TNF- α were significantly higher in malaria-infected individuals compared to non-infecting individuals and cytokine levels were found to be correlated with recurrent malaria infection.

## Introduction

Malaria is a mosquito-borne disease that affects humans and animals. This condition is caused by a protozoan parasite of the genus *Plasmodium*. Malaria symptoms include fever and headache, which result from parasite invasion of red blood cells. In severe cases, malaria infection may progress to coma or death ( Elmardi *et al*., 2011). There are five *Plasmodium* species that are known to cause disease in humans ( *P. falciparum*, *P. vivax*, *P. ovale*, *P. malariae*, and the recently described *P. knowlesi*). The species that causes the most severe cases is *P. falciparum*. Malaria control efforts in Sudan began in 1904, when Dr. A. Balfour succeeded in eradicating malaria from Khartoum ( Malik *et al.,* 2006). Determination of the correct prevalence of malaria is essential in implementing effective control strategies to curb its dissemination ( Malik *et al.,* 2006).

Immunity to *Plasmodium* develops slowly and protection against *Plasmodium* occurs later than protection against malaria symptoms. The immune responses will not be same for the liver and blood stages because *Plasmodium* expresses various antigens at the liver and blood stages ( Langhorne, 2005). Cytokines may assume a significant role in protection and pathology in malaria. The early effective inflammatory reaction is regulated by IFN-γ, IL-12. TNF- α appears to be crucial for parasitemia control in malaria infection ( Artavanis-Tsakonas *et al*., 2003). However, pro-inflammatory cytokines (TNF- α, IFN-γ, IL-1 and IL-6) were associated with severe malaria ( Malaguarnera & Musumeci, 2002). The high production of proinflammatory cytokines may increase cytoadherence of parasitized red blood cells to the endothelium through upregulation of adhesion molecules in *P. falciparum* infections ( Day *et al.,* 1999).

The expression of cytokines (pro- and anti-inflammatory) are said to be involved in malaria pathogenesis. Severe malaria has been associated with low serum levels of IL-12 and low IL-10 to TNF- α serum concentration ratios in a few studies of childhood malaria in holoendemic areas.

In order to explore the effect of the immune response to malaria and the development of clinical immunity, this study aimed to measure and determine the prevalence of malaria and quantify cytokine levels in patients with malaria infection in the East Nile locality.

## Methods

This cross-sectional study was carried out in the East Nile locality, which is located in the eastern part of Khartoum State, Sudan. This study was conducted in different pre-urban areas during the period from 1 ^st^ May to 23 ^rd^ of July 2018.

### Study population

The study population included participants of all ages and genders from a population admitted to Elbanjadeed Hospital, Aldebaba Medical Health Center, Eid Babekir Medical Health Center, Helat Koko Medical Health Center and Omdom Medical Health Center. In total, 384 participants were asked to participate in this study, all of whom were admitted for malaria diagnosis. Participants were selected using quota sampling and all patients admitted for malaria diagnosis were eligible to be included in the study. The age groups were categorized as follows: less than 10 years, 11–49 years and over 50 years old. After participants signed an informed consent form for participation in the study, a questionnaire was used by expert laboratory technician to collect demographic data (information about age, sex, residence and occupation) and medical history of chronic disease (renal disease, heart disease and diabetes mellitus or other chronic disease) from patients enrolled in the study.

### Sample size

In total, 384 samples were collected from patients admitted for malaria infection at hospitals and health centers. The sample size was calculated on the following formula ( Daniel, 1999):

$$\text{N}={\text{Z}}^{\text{2}}\text{p}(1-\text{p})/{\text{d}}^{2}$$

where N = sample size, Z = statistic for a level of confidence (1.96), P = prevalence in study area (50%) and d = precision (5%).

### Sample collection

From each patient, thick and thin blood films were prepared using finger prick blood samples taken as part of the routine diagnosis of infection with *Plasmodium* species. Thin films were fixed with methanol and slides were placed face down on a drying rack for five minutes to allow the methanol to fix. Thick and thin blood films were stained with Giemsa stain at a concentration of 10% for 10 minutes. The stain was flushed from the slides by adding drops of buffered water until all the stain has been washed away. Where the blood film analysis was positive for malaria, 5 ml of venous blood was collected from each patient into a sterile container. For cytokine analysis, 5ml of venous blood was also collected from 10 participants who tested negative for malaria and agreed to participate further in the study. These participants were selected using stratified random sampling, with two participants being randomly selected from each of the five health centers. Following collection, blood samples were centrifugated at 3000 rpm for 10 minutes. After centrifugation, the serum was separated and transferred to another labeled sterile container and stored in refrigerator at 4 ^o^C until use.

### Microscopic examination

After the films dried, they were examined microscopically by experienced personal to determine the parasite stages (ring, trophozoite, gametocyte and schizont), using the thin blood film to identify the species of *Plasmodium* and the thick film to classify parasitemia as follows:

+: 1–10 parasites per 100 thick film fields
  + +: 11–100 parasites per 100 thick film fields
  + + +: 1–10 parasites per one thick film field
  + + + +: more than 10 parasites per one thick film field

### Measurement of cytokines

For cytokine analysis, 29 of the patient serum samples were selected using simple stratified sampling, with five positive samples randomly selected from each health center and nine randomly selected from the hospital. Cytokine analysis was not performed for all samples due to financial restrictions. The stored serum was brought to the laboratory and was allowed to thaw. Serum concentrations of IFN-y, IL-10, TNF-α were determined using an enzyme-linked immunosorbent assay (ELISA) according to manufacturer’s instructions (BioLegend’s ELISA MAX™ Deluxe Sets, catalog numbers 430104, 430604, and 340204 for IFN-y, IL-10, and TNF-α, respectively) for the patient samples and 10 control samples.

Briefly, 100µL of diluted capture antibody solution was added to each well and the sealed plate was incubated overnight between 2–8°C. The plates were washed four times and then blocked by adding 200µL assay diluents to each well, then were sealed and incubated for one hour with shaking on a palate shaker at 500 rpm with a 0.3cm circular orbit. The plates were washed four times and then 100μl diluted standards and samples were added to each well. The plate was sealed and incubated at room temperature for two hours with shaking. The plate was washed four times, then 100µL diluted detection antibody solution was added to each well. Plates were sealed and incubated at room temperature for one hour with shaking. The plate was washed four times, then 100μl diluted avidin–HRP solution was added to each well. The plate sealed and was incubated at room temperature for 30 minutes with shaking. The plate was washed five times and then soaked for 30 seconds to one minute per wash. Then, 100µL fresh TMB substrate solution was added to each well and incubated in the dark for 20 minutes. Finally, 100 µL of stop solution was added to each well and the absorbance was read with the SPECTROstar Nano Microplate Reader at 540 nm and 570 nm within 15 minutes.

### Statistical analysis

Data were analyzed using SPSS version-20. The Chi-squared test was performed to determine statistical significance and a *P*-value of less than 0.05 was considered statistically significant.

### Ethical statement

Ethical clearance for this study was obtained from Committee of Scientific Research Deanship, Sudan University of Science and Technology, ethical approval number (DSR – IEC – 12 – 07). Written informed consent for participation and publication of the data was obtained from all participants included in this study or for children, from their guardian.

## Results

A total of 384 patients were enrolled in this study, of which 154 were male and 230 were female. Their ages were grouped into three categories: less than 10 years old (134), 11 – 49 years old (198) and more than 50 years old (52).

### Prevalence of malaria in patients from the study area

First, we sought to determine the prevalence of malaria in our study population. To this end, we collected blood samples from our patients and examined them using blood films. Out of 384 blood samples collected from different pre-urban areas (medical centers and hospitals) in the East Nile locality during the period from May to July 2018 (pre- malaria season), 71 (18.5%) were found to be positive and 313 (81.5 %) were negative for malaria ( Table 1) ( Abd Alla & Brakat, 2019). Moreover, we observed a higher prevalence of malaria among males (22.7%) compared to females (15.6%) in our study population ( Table 1). In addition, analysis of the prevalence among different age groups revealed that highest prevalence rate was in the under 10 age group (20.1%), followed by the 11–49 age group with a prevalence of 19.7%, while the lowest prevalence rate was reported among the over 50 age group (2%) ( Table 1).

**Table 1.** Overall prevalence of malaria in study area, assessed using blood films.

Blood film	Frequency	Percentage
Positive	71	18.5
Negative	313	81.5
Total	384	100.0
Gender	Number examined	Number positive (%)
Male	154	35 (22.7)
Female	230	36 (15.6)
Total	384	71 (18.5)
Age group		
≤ 10	134	27 (20.1)
11 – 49	198	39 (19.7)
≥ 50	52	5 (2)
Total	384	71 (18.5)

### Distribution of *Plasmodium* species in the study area

Next, we determined the species distribution of *Plasmodium* species in our study population. We observed that *P. falciparum* had the highest prevalence rate (13%), followed by *P*. *vivax* (4.6%). However, mixed infection by *P. falciparum* and *P. vivax* had the lowest prevalence rate (0.8%). We failed to detect any positive results for *P. malariae* or *P. ovale* infection in our study population ( Table 2).

**Table 2.** Distribution of *Plasmodium* species in the study area.

Species	Frequency (%)
*P. falciparum*	51 (13)
*P. vivax*	17 (4.6)
*P. ovale*	0 (0)
*P. malariae*	0 (0)
*P. falciparum and P. vivax* co *-*infection	3 (0.8)
Total	71 (18.5)

Our data reveal that ,there was no significant statistical association between P.falciparum and *P.vivax* in cytokines level, P.value were (0.436, 0.789 and 0.530) for INF – γ , TNF-α and IL-10 respectively.

### Severity of malaria infection among the study population

Next, we determined the distribution of severe malaria in the studied population. Our data showed that most (58%) of the study population had a low parasite count (mild parasitemia), while 13% had a moderate parasitemia (++). We detected no cases that exhibited severe parasitemia (+++ and ++++) ( Table 3) and no statistically significant association between age group and parasitemia ( Table 4).

**Table 3.** Prevalence of malaria according to parasite count.

Parasite count	Frequency (%)
+	58 (81.7)
++	13 (18.3)
+++	0 (0)
++++	0 (0)
Total	71 (18.5)

**Table 4.** Correlation between parasitemia and age.

Density	Age group (%)			Total	*P* value
	≤ 10	11–49	≥ 50		
+	20(34.5)	31(53.4)	7(12.1)	58	0.108
++	7(53.8)	6(46.2)	0	13	
+++	0	0	0	0	
++++	0	0	0	0	

### Cytokine profiles in malaria-infected patients

Cytokines may play a role in protection and pathology in malaria. Thus, we investigated the cytokine profile in 29 patients and 10 controls from our study population. In particular, we investigated serum levels of IFNγ, TNF-α, and IL-10 with malaria infection in the study population. Interestingly, mean serum levels of IFNγ were significantly higher in malaria-infected individuals compared to non-infected individuals ( *P* value = 0.026). However, TNF-α serum levels were comparable between patients and non-infected individuals ( *P* value = 0. 646). Mean serum levels of IL-10 were higher in patients compared to non-infected individuals, although this difference was not statistically significant ( *P* value = 0.071) ( Table 5).

**Table 5.** Cytokine profiles of the study population.

Sample	Number	Cytokine profiles (mean ± SD)		
		IFN-γ (ng/ml)	TNF-α (ng/ml)	IL-10 (ng/ml)
Patients	29	61.98 ± 71.93	5.91 ± 5.67	48.87 ± 52.99
Controls	10	11.05 ± 11.05	5.36 ± 3.78	12.48 ± 8.08
*P* value		0.026	0.646	0.071

Next, we sought to determine whether there is a correlation between the cytokine profiles of individuals enrolled in our study and severity of malaria and/or recurrent infection. However, we found no correlation between levels of IFNγ, TNF-α, or IL-10 and level of parasitemia ( Table 6). Intriguingly, levels of TNF-α and IL-10 were significantly higher in patients who suffered from recurrent malaria infection compared to those who did not ( Table 7). However, we failed to detect a significant correlation between levels of IFNγ and recurrent malaria infection ( Table 7).

**Table 6.** Correlation between parasitemia and cytokine profiles.

Density	Number	Mean		
		IFN-γ (ng/ml)	TNF-α (Ng/ml)	IL-10 (Ng/ml)
+	25	79.49	5.80	60.50
++	4	79.94	7.98	67.17
*P* value		0.922	0.525	0.380

**Table 7.** Correlation between recurrent malaria infection and cytokine profiles.

Recurrent malaria infection (yes/no)	Number	Mean (ng/ml)	SD (ng/ml)	*P* value
*IFN-γ*				
Yes	15	83.06	70.61	0.151
No	24	48.82	71.02	
*TNF-α*				
Yes	15	8.76	8.10	0.011
No	24	4.13	2.03	
*IL-10*				
Yes	15	77.21	65.55	0.007
No	24	31.16	34.26	

## Discussion

The findings of our study revealed a prevalence rate of malaria of 18.5%. This rate was greater than the rate reported in Khartoum by El Mekki *et al.* (2012), who reported the prevalence of malaria in 5% and 11% in Dar Al Salam Camp and Jabal Awlia Camp, respectively. El Sayed *et al.* (2000) reported that Khartoum, which was formerly malaria free, can be considered as a hypoendemic or mesoendemic area in which malaria is unstable and epidemic outbreaks are common; our results agree with their findings.

Our study show that Falciparum malaria is the most prevalent and constitutes about 13% of all infections, benign tertian Vivax malaria has prevalence of 4.6%, and the lowest prevalence rate of 0.8% is observed for mixed infection ( *P. falciparum* and *P. vivax*). However, we observed no cases of *P. malariae* and *P. ovale* infection. Moreover, males had higher prevalence rate (22.7%) of malaria infection than females (15.6%). Our study findings agree with a study in Khartoum by Abdalla *et al.* (2007), who reported that the overall prevalence of malaria was 28.2 % and was higher in males than in females.

The highest prevalence rate (53.8%) of moderate parasitemia was in the under 10 years age group. Although children are more susceptible to malaria infection due to a slow developing immune system, a high prevalence rate (34.5%) of mild parasitemia was reported among the 11–49 years age group. A lower prevalence rate of 2% and mild parasitemia 12.1% was reported among the over 50 age group. This finding was closer to the finding of Igwe *et al.* (2014) in Nigeria, who reported that the highest prevalence of asymptomatic malaria parasitemia (87.5%) was found in parturient women who were ≤19 years, while the lowest prevalence (68.2%) occurred in those who were 40–49 years old. In the present study we observed that age group was not significantly associated with parasitemia. This is in agreement with a study done by El Khalifa *et al*. (2008), who found no significant difference in parasitemia among those aged five years and above.

In this study, serum levels IFN-γ, TNF- α and IL-10 were measured in healthy controls and in patients with *P. falciparum* and *P. vivax* infection and IFN-γ was found to be significantly higher in patients than in non-infected individuals. This finding is in line with a study in Poland by Wroczynska *et al.* (2005), who reported that the mean serum level of IFN-γ was found to be significantly higher in severe and uncomplicated malaria groups compared to the controls. Also, another study done by Favre *et al.* (1997) reported that these findings are consistent with a requirement for an early production of IFN-γ to mount resistance against infection. Interestingly, in this study, a significant correlation between IL-10 with gender and age was found. This association between initial IL-10 levels and parasite densities agreed in part with the findings of Hugosson *et al*. (2004), who reported similar findings during patient treatment, indicating that IL-10 levels may play a role in clearance of parasites during treatment. In addition, they also suggest that there are age-related differences in immunity and the development of partial clinical tolerance.

The present study reported no association between parasite density and levels of IFN-γ, TNF-α and IL-10. These findings are in line with study done by Jason *et al*. (2001) in which serum IL-10 levels had statistically significant association with level of parasitemia. Furthermore, our data agrees with a study by Nnaemaka *et al.* (2009), who found no significant correlation between IL-10, IL-12 and IFN-γ in asymptomatic individuals with parasitemia; however, Wroczynska *et al*. (2005) found that IL-10 and IL-12 were associated with malaria. In this study, there was significantly increased production of IL-10 and TNF-α in patients with recurrent malaria. This finding was in agreement with a study done by Edward *et al*. (2008), who reported that the high levels of IL-10 observed during malarial episodes may be beneficial, acting to reduce the inflammatory response. However, they may also be detrimental and decrease antiparasitic cellular immune responses. This is suggested by our data, with significant levels of TNF- α found in patients with recurrent malaria infection. This is in line with Medzhitov *et al*. (2012), who reported that these observations are predictable, with the possibility that recurrent malaria may drive the host towards a disease tolerant state, so as to diminish the negative effects of disease-related pathology. In subjects who are routinely infected by malaria, the pro-inflammatory response may be immediately controlled by regulatory mechanisms. This impact might be particularly exaggerated in this study area, where transmission is particularly extreme.

## Data availability

### Underlying data

Figshare: hoda datta.sav. https://doi.org/10.6084/m9.figshare.8986178.v2 ( Abd Alla & Brakat, 2019)

This project contains the following underlying data:

- hoda datta.sav (demographic, behavioral and medical data for each participant and results of the microscopic examination)
  - samle and control.sav (cytokine levels for 39 patient and control samples, determined using ELISA)
  - Data dictionary_FL.docx

Data are available under the terms of the Creative Commons Zero “No rights reserved” data waiver (CC0 1.0 Public domain dedication).
