# Supplementary material for: Prevalence of malaria and quantification of cytokine levels during infection in East Nile locality, Khartoum State: a cross-sectional study
Source: F1000Res. 2021 Aug 16;8:1529. Originally published 2019 Aug 29. [Version 2] doi: 10.12688/f1000research.19217.2 (PMC8442113; doi:10.12688/f1000research.19217.2)
Supplement: Peer review history

# Peer review: Reviewer response for version 2

**DOI:** 10.5256/f1000research.76573.r92038

**Published:** 2021-09-14

**Authors:** Rusliza Basir

**Affiliations:** 1 Department of Human Anatomy, Faculty of Medicine and Health Sciences, University Putra Malaysia (UPM), Serdang, Malaysia

The authors have attended to all the comments satisfactorily.

Is the work clearly and accurately presented and does it cite the current literature?

Yes

If applicable, is the statistical analysis and its interpretation appropriate?

Yes

Are all the source data underlying the results available to ensure full reproducibility?

Yes

Is the study design appropriate and is the work technically sound?

Partly

Are the conclusions drawn adequately supported by the results?

Partly

Are sufficient details of methods and analysis provided to allow replication by others?

Yes

Reviewer Expertise:

Immunotherapeutic target for malaria infection.

I confirm that I have read this submission and believe that I have an appropriate level of expertise to confirm that it is of an acceptable scientific standard.

# Peer review: Reviewer response for version 2

**DOI:** 10.5256/f1000research.76573.r92037

**Published:** 2021-08-20

**Authors:** Yaowapa Maneerat

**Affiliations:** 1 Department of Tropical Pathology, Faculty of Tropical Medicine, Mahidol University, Bangkok, Thailand

I confirm that I have read the revised article carefully. The authors have already corrected some points according to my suggestion. I approve the revised article. However, I feel that Table 2 and 3 (with only 2 columns) are not necessary. If possible, the authors should describe completely using 2-3 sentences.

Is the work clearly and accurately presented and does it cite the current literature?

Yes

If applicable, is the statistical analysis and its interpretation appropriate?

Partly

Are all the source data underlying the results available to ensure full reproducibility?

Partly

Is the study design appropriate and is the work technically sound?

Partly

Are the conclusions drawn adequately supported by the results?

No

Are sufficient details of methods and analysis provided to allow replication by others?

Yes

Reviewer Expertise:

Pathology of malaria

I confirm that I have read this submission and believe that I have an appropriate level of expertise to confirm that it is of an acceptable scientific standard.

# Peer review: Reviewer response for version 1

**DOI:** 10.5256/f1000research.21057.r66205

**Published:** 2020-07-03

**Authors:** Rusliza Basir

**Affiliations:** 1 Department of Human Anatomy, Faculty of Medicine and Health Sciences, University Putra Malaysia (UPM), Serdang, Malaysia

1. It was mentioned that patients serum samples were selected randomly for cytokines analysis. How does this random selection carry out? Does it take into consideration the age of patients since they are categorized according to age at the beginning? There would be differences in cytokine release profile between child, adult and elderly as the level of immune response between the three groups towards invading organisms would be different. So how does the selection carry out among the three groups of patients?
2. The symbol for gamma for IFN must be presented properly with the right symbol… not “y”.
3. What is the age profile or category of the 29 patients in whom the cytokine profile was carried out? It would be interesting to see if the cytokines release is influenced by age.

Is the work clearly and accurately presented and does it cite the current literature?

Yes

If applicable, is the statistical analysis and its interpretation appropriate?

Yes

Are all the source data underlying the results available to ensure full reproducibility?

Yes

Is the study design appropriate and is the work technically sound?

Partly

Are the conclusions drawn adequately supported by the results?

Partly

Are sufficient details of methods and analysis provided to allow replication by others?

Yes

Reviewer Expertise:

Immunotherapeutic target for malaria infection.

I confirm that I have read this submission and believe that I have an appropriate level of expertise to confirm that it is of an acceptable scientific standard, however I have significant reservations, as outlined above.

# Peer review: Reviewer response for version 1

**DOI:** 10.5256/f1000research.21057.r58145

**Published:** 2020-01-16

**Authors:** Yaowapa Maneerat

**Affiliations:** 1 Department of Tropical Pathology, Faculty of Tropical Medicine, Mahidol University, Bangkok, Thailand

As per my knowledge, there have been several studies in roles of cytokine profiles in human and animal malaria. This study aimed to determine important cytokines in malaria. These included IFN-γ, IL-12. TNF- α. They used rather appropriate sample size. However, as follows, it was found some points are unclear and should be clarified. 

1. The conclusion in the abstract is not clear. The authors did not indicate any cytokines.
2. The rationale and the benefit of study is not clear.
3. The author did not show the difference in any cytokine levels between *P. falciparum* and *P. vivax* infected patients.
4. It seems that there are too many tables. Is it possible to merge Table 1 and 2; 3 and 6?
5. Correlation between parasitemia and cytokines in Table 8 is not reliable. Sample size (n=4) is too small for determining correlation.
6. The authors did not mentioned about detail of patients in ELISA results. Why sample size was 10 control and 29 patients?

Is the work clearly and accurately presented and does it cite the current literature?

Yes

If applicable, is the statistical analysis and its interpretation appropriate?

Partly

Are all the source data underlying the results available to ensure full reproducibility?

Partly

Is the study design appropriate and is the work technically sound?

Partly

Are the conclusions drawn adequately supported by the results?

No

Are sufficient details of methods and analysis provided to allow replication by others?

Yes

Reviewer Expertise:

Immunopathology in malaria.

I confirm that I have read this submission and believe that I have an appropriate level of expertise to confirm that it is of an acceptable scientific standard, however I have significant reservations, as outlined above.
